# A Novel Bike-Mounted Sensing Device with Cloud Connectivity for Dynamic Air-Quality Monitoring by Urban Cyclists

**DOI:** 10.3390/s22031272

**Published:** 2022-02-08

**Authors:** Jaime Gómez-Suárez, Patricia Arroyo, Raimundo Alfonso, José Ignacio Suárez, Eduardo Pinilla-Gil, Jesús Lozano

**Affiliations:** 1 Escuela de Ingenierías Industriales, Universidad de Extremadura, 06006 Badajoz, Spain; jaimegs@unex.es (J.G.-S.); parroyoz@unex.es (P.A.); jmarcelo@unex.es (J.I.S.); 2 Ray Ingeniería Electrónica, 10540 Mirabel, Spain; ray@ray-ie.com; 3 Facultad de Ciencias, Universidad de Extremadura, 06006 Badajoz, Spain; epinilla@unex.es

**Keywords:** air-quality monitoring, low-cost sensors, sensor calibration, electrochemical gas sensor, optical particle counter

## Abstract

We present a device based on low-cost electrochemical and optical sensors, designed to be attached to bicycle handlebars, with the aim of monitoring the air quality in urban environments. The system has three electrochemical sensors for measuring NO_2_ and O_3_ and an optical particle-matter (PM) sensor for PM_2_._5_ and PM_10_ concentrations. The electronic instrumentation was home-developed for this application. To ensure a constant air flow, the input fan of the particle sensor is used as an air supply pump to the rest of the sensors. Eight identical devices were built; two were collocated in parallel with a reference urban-air-quality-monitoring station and calibrated using a neural network (R^2^ > 0.83). Several bicycle routes were carried out throughout the city of Badajoz (Spain) to allow the device to be tested in real field conditions. An air-quality index was calculated to facilitate the user’s understanding. The results show that this index provides data on the spatiotemporal variability of pollutants between the central and peripheral areas, including changes between weekdays and weekends and between different times of the day, thus providing valuable information for citizens through a dedicated cloud-based data platform.

## 1. Introduction

In recent years, air quality has become a major problem in large cities. Although episodic increases in pollutant levels can be caused by natural events such as volcanoes (e.g., SO_2_) or desert dust outbreaks (PM), the main emission sources of pollutant gases and particulates are of human origin, such as traffic and industrial activities [1]. As demonstrated by scientists, notably by World Health Organization (WHO) expert groups, these compounds can lead to respiratory problems. In particular, they have been linked to the appearance of lung cancer and they contribute to the appearance of mental disorders [2]. These reasons have prompted governments of large cities and national and international organizations to take surveillance and control measures to prevent the impact of atmospheric pollution, which often involve novel clean technologies and traffic restrictions.

The process of obtaining and processing pollutant data to monitor and assess air quality has been separated into three stages by some authors: monitoring, prediction, and tracing [3]. Currently, regulatory monitoring is carried out by large and expensive analytical equipment based on standard and certified measurement techniques that is installed inside fixed or mobile (large vehicles) reference stations placed at points of special interest. These stations and the equipment they contain have a high installation cost and require periodic maintenance by qualified personnel. Moreover, they require an extensive energy supply for air conditioning (operating temperature 20 °C) and instrument operation. Aside from these restrictions, the monitoring strategy based on sophisticated fixed and mobile stations involves an additional problem, which is the very limited spatial resolution of the data that are provided, which compromises the representativeness of the estimated risk to human health and ecosystems.

In this scenario, low-cost sensors (LCSs) have emerged in recent years as complementary tools that, combined with conventional equipment, allow air quality to be monitored more effectively, dramatically improving the spatial resolution of air-monitoring data and effectively engaging citizens in the experimental measurements [4]. These sensors have much lower price and energy requirements than conventional equipment. As described in [5], the price of low-cost sensors is between $10 and $100, which increases to about $1000 to $5000 when the sample supply, electronic systems, protective housing, connections, and management hardware and software are included. LCSs can be installed in easily available locations (typically urban posts, trees, balconies, etc.), worn on vehicles (such as bicycles), or carried by citizens, thereby improving the spatial and temporal representativeness of pollutant-level measurements [6]. Moreover, a large number of LCSs installed in a given area allows a high spatial resolution to be obtained in that area, as exemplified by two studies, one that monitored the air quality of the Gold Coast (Australia) for several weeks [7] and one using a network of 40 nodes to control air quality at Heathrow Airport (London) [8].

Low-cost gas sensors can be classified according to their principle of operation. In this way, a distinction is made between electrochemical, metal-oxide (MOX), and optical sensors such as non-dispersive infrared (NDIR) and optical particle-counter (based on light dispersion) sensors. For particulate-matter measurement, the most common sensors are based on photometry using a scattering laser, although there are also some based on gravimetric sampling and beta attenuation [9].

Once calibrated in the lab, LCSs need to be validated for field use in real conditions. In addition, low-cost sensors often present interference between contaminants (cross-sensitivity), especially between NO_2_ and O_3_, which should be evaluated and corrected during real operation. On the other hand, they are also affected by environmental conditions, especially temperature and relative humidity [10,11], which are not controllable variables when working in the field. Finally, these devices have drift, i.e., the response of the sensor is affected as it is working. Due to all of these aspects, it is common in the literature to see different approaches to calibrating these sensors and obtaining reliable data from them, mostly based on machine-learning algorithms such as neural networks, linear regressions, or support vector machines. For example, in [12] these algorithms were used to study the air inside a running vehicle. In [13], the authors obtained good results using a long short-term memory network. In [14], the authors used a different approach; they first conducted a linear regression and then introduced the result into a neural network. Most authors agree that the quality of the LCS data is good enough if the data fulfill the objective of providing useful air-quality information to citizens, or if the objective is to distinguish between different levels of pollution on a semiquantitative basis (high, low, moderate, etc.). Providing citizens with a simple and easy-to-understand system for estimating air quality on a local scale is thus mandatory and was one of the main purposes of this work.

This work is framed within the NanoSenAQM project, a European initiative that pursues the monitoring of air quality by designing, fabricating, and testing a range of LCSs in different natural, rural, and urban environments [15]. One of the main contributions of this work is the device presented, which is designed to be installed on bicycle handlebars in order to monitor air quality and its dynamic spatiotemporal evolution. The novelty of our air-quality-monitoring strategy also lies in the implementation of multiparametric neural-network calibration coupled with cloud connectivity and air-quality-index calculation, which readily allows cyclists to get a clear overview of their degree of exposure to air pollution during urban travel, providing a basis for personal decisions such as route selections or preferable time slots. It is also valuable information for urban designers, e.g., for the planning of bike paths. We consider it more refined and personalized information than the data provided by smartphone apps based on reference air-quality-monitoring units, which are sometimes located at quite remote points with respect to the user’s location. Our device has been tested on bicycles, but it can be easily adapted to different vehicles and even for static usage. As shown in [16], it is much more common to find LCSs designed for operation at fixed points, or coupled to UAVs in case a mobile device is needed, than attached to bicycles. However, drones must comply with flight rules, so they are not the best choice if the objective is to study the air quality in an urban area at low altitude.

In the following sections of this work, we describe in depth the device that was designed for this task, the calibration and field validation and the measurement campaigns, including the problems found during the experimental work. The most relevant results are shown and discussed and, finally, the work concludes with the most relevant ideas obtained.

## 2. Materials and Methods

### 2.1. Description of the Device

**Figure 1:**
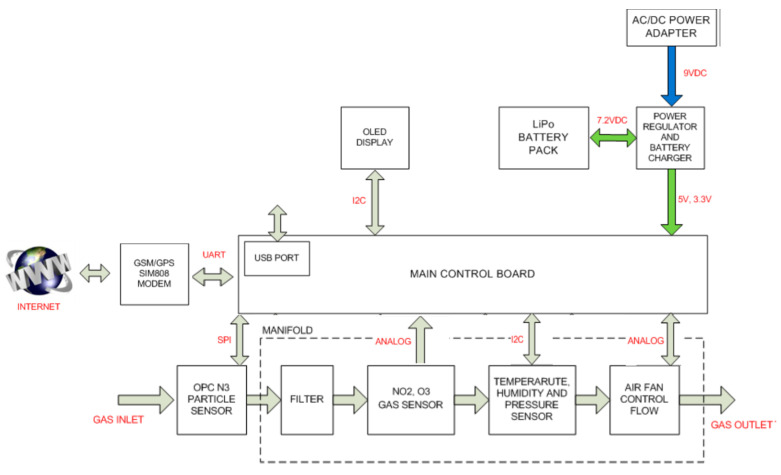
General diagram of device.

The device, described in Figure 1, was developed entirely and exclusively to measure the main pollutants (NO_2_, O_3_, PM_2_._5_, and PM_10_) responsible for worsening air quality. The prototype was designed to fit on a bicycle in order to provide increased spatiotemporal resolution of pollutant maps and estimate the air quality perceived by cyclists. That is why great importance was given to optimizing the device to allow its installation and operation on bicycles, with special attention paid to the autonomy, size, weight, and communication method implemented.

The air-quality-sensor array is composed of 3 sensors produced by AlphaSense (Essex, UK). Two are A4 series 3-electrode electrochemical sensors designed to measure NO_2_ and O_3_. The third is an OPC-N3 optical particulate sensor that provides PM_10_ and PM_2_._5_ concentration values by light dispersion, using an internal algorithm.

In addition to these 3 sensors, the device has a number of sensors focused on monitoring environmental conditions, which can provide relevant information when the signals are processed. These include a temperature, pressure, and relative-humidity sensor (BMP280) from Bosch Sensortech. The values of the gas sensors are read by the microcontroller from the built-in 12-bit A/D conversion inputs. The particle sensor uses SPI communication. Finally, the pressure, temperature, and humidity sensor use the I^2^C bus.

The complete system is controlled by a low-power, high-performance ARM^®^ Cortex^®^-M0+-based flash microcontroller (Microchip ATSAMD21G18), which is a 32-bit microcontroller with an operating frequency of up to 48 MHz. It is connected via I^2^C interface to an OLED display that shows the sensor values and the operating status of the device (battery, date, time, temperature, GPS status, etc.). The system carries out communication in different ways. First, the GSM/GPRS module (SIM808, SIMCom) allows wireless communication with the internet to store information coming from the sensors in the cloud. This module communicates with the microcontroller via UART connection. There is also a USB input for manual data transfer and device programming. Finally, all of the collected information is also stored locally on a microSD card: date, time, latitude, longitude, altitude, number of satellites in coverage and used, battery voltage and percentage, temperature, humidity, pressure, external-fan rpm, PM_1_, PM_2_._5_, PM_10_, OPC-N3 sensor flow rate, NO_2_ (ppb), O_3_ (ppb), and the raw values of the gas-sensor electrodes. This study focused on NO_2_, O_3_, PM_2_._5_ and PM_10_. Information is stored in a text file (.txt) with a sampling period of 3 s.

The system is powered by a lithium-ion battery with a capacity of 2750 mAh, which provides system autonomy of up to 8 h. In addition, a jack connector is incorporated to charge the system from a 9 V, 660 mA power supply.

The pneumatic sampling system of the whole sensing device uses the OPC-N3 sensor as the input, since it has an air inlet with flow control from a fan. The air is conducted to a collector, where the rest of the sensors are located, and finally it is expelled to the outside by a second fan placed at the outlet. It should be noted that this collector includes a conductive coating; in addition to redirecting the air flow through the gas sensors, it also protects the sensors against electromagnetic interference.

**Figure 2:**
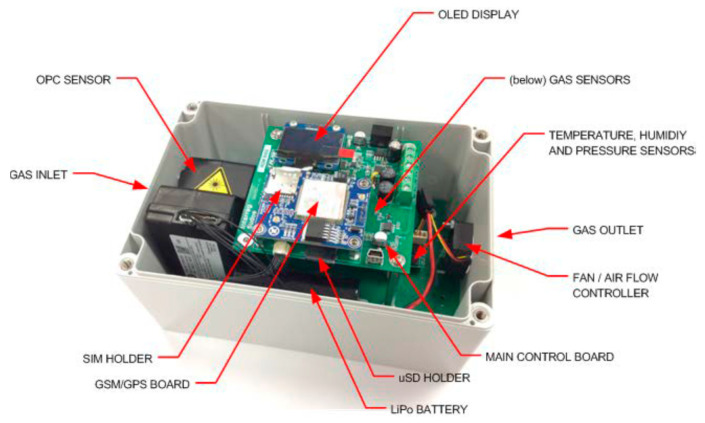
Device inside protective casing.

All of these elements are protected inside a polycarbonate housing with IP66 protection with dimensions of 180 × 120 × 90 mm, which can be seen in Figure 2. The housing is attached to the handlebars of a bicycle by an adjustable adapter designed to fit most models. The approximate weight of the complete system is 1.1 kg, which is appropriate for use without disturbing the cycling experience of the user. Nevertheless, we suggest that a reduced size and weight are desirable technical aspects to be considered in further developments.

With this design, eight identical devices were manufactured, and two of them, coded BEC01 and BEC02, were used in the experiment. The first one served as the object of study, while the second one was used to study the repeatability of the design, which is detailed in Section 3.3.

### 2.2. Gas Sensors and Particulate Matter Sensor

Two electrochemical sensors manufactured by AlphaSense, NO2-A43F and OX-A431, were used to measure concentrations of NO_2_ and O_3_, respectively. As shown in [17], electrochemical gas sensors are the most commonly used type of LCS for air-quality monitoring and have demonstrated good performance (R^2^ = 0.90 and R^2^ = 0.81 for NO_2_ and O_3_, respectively, when calibration with an artificial neural network was performed). These sensors calculate the concentration of a given gas based on the changes it produces in the electrical properties of an electrode. The sensors are composed of 4 electrodes: working, reference, counter, and auxiliary electrodes [18]. From the current of the working and auxiliary electrodes, the gas concentration is calculated by means of a conversion algorithm, which will be detailed in Section 2.5.1.

The OPC-N3 sensor was chosen to measure the particle concentration. It is an optical particle counter (OPC) that uses Mie scattering to estimate the concentrations of particulate matter in the air [19]. The sensor incorporates a small fan that draws air inside, where a laser beam passes through the sample and strikes the suspended particles. Knowing the intensity of the scattered light and the refractive index, it is possible to estimate the PM concentration.

### 2.3. Measurement Campaigns

To fit and experimentally test the prototypes, the measurement campaign was conducted in two parts: first, calibration and validation of the sensors, and second, testing of the devices coupled to a bicycle performing several routes on different days. The place chosen for both the calibration and testing was the city of Badajoz (Spain).

#### 2.3.1. Calibration Measurements

Calibration by adjusting the sensor measurements to the reference instrumentation (see details in Section 2.5) was carried out from 18 to 20 January 2021. During that period, the devices were collocated in parallel with a reference station at an urban location with high traffic density (38°52′14.6″ N 6°58′43.6″ W) to ensure that the sensors worked within a wide concentration range and with the high temporal variability typical of urban traffic. The air-quality-monitoring instruments that were used as a reference belonged to the Air Quality Protection and Research Network of Extremadura (REPICA), of the Department of Ecological Transition and Sustainability of the Regional Government of Extremadura. The devices were installed in the same position at which they were subsequently placed on the bicycles; thus, the conditions were similar in both scenarios.

The reference equipment used was as follows:

O_3_: Thermo Fisher 49i-B3ZAA (UV absorption)

NOx: Thermo Fisher 42i-BZMTPAA (chemiluminescence)

PM: DIGITEL DHA-80 (high-volume sampler + gravimetric analysis) and GRIMM 180 (optical laser light aerosol spectrometer, non-regulatory)

Data collected by the reference system, comprising 10-min-average values in concentration units of micrograms per cubic meter (μg/m^3^), were validated before being used for calibration. Data from the developed devices were averaged over the same intervals for comparison. This information was used to apply the calibration techniques described in Section 2.5.

#### 2.3.2. Cycling Routes

To test the devices in the field, three routes (R1, R2, and R3), consisting of 90–120 min bicycle rides through Badajoz were carried out on 22, 24, and 28 January 2021. During these routes, the bicycle travelled both on roads with high traffic density and in quiet areas with more vegetation and less traffic impact. In addition, the bicycle was equipped with a camera to record the entire route, in order to have more information when analyzing the results. The data collected by the sensors were corrected using the calibration algorithm. The air-quality index was calculated and the results were mapped, as shown in Section 3.4.

### 2.4. Data Acquisition and Filtering

The devices store pollutant-concentration data, data on the gas-sensor electrodes, the air flow of the OPC-N3 sensor, and data related to the environment on a microSD card, along with the device status such as temperature, humidity, battery, latitude, longitude, and altitude, with a sampling frequency of 3 datapoints per second. The data are then averaged to obtain an appropriate resolution, and all units are converted to micrograms per cubic meter (μg/m^3^) for later study.

In addition, the devices send the concentration data to a cloud platform developed by the University of Coimbra and the University of Evora within the framework of the European NanoSen-AQM project [20,21]. This platform was developed so that users can have access to the collected data and accurate information on the quality of the air around them.

### 2.5. Calibration Process

#### 2.5.1. Internal Algorithms

The gas sensors use the value of the working and auxiliary electrodes to calculate pollutant concentrations using an internal algorithm designed by the manufacturer through the following expression:

(1) $$\left[Pollutant\;\right]\left(\mathrm{ppb}\right)=\frac{\left(S_{WE}-S_{WE,0}\right)-n\left(S_{AE}-S_{AE,0}\right)}{s}$$

where *S_WE_* and *S_AE_* are the values of the working and auxiliary electrodes, respectively; *S_WE_*_,0_ and *S_AE_*_,0_ are the offset of the working and auxiliary electrodes; n is a temperature-dependent parameter given by the manufacturer; and *s* is the sensitivity to the contaminant.

This calculation of the concentration from the voltage values was designed for stable conditions and indoor applications. Several authors [22,23] have shown that if the objective is to improve the performance of the sensors when working outdoors, then it is more advisable to work with the electrode values (*S_WE_* and *S_AE_*) than with the concentration values calculated from Formula (1).

Optical particle counters (OPCs) are calibrated in the laboratory by the provider, relating the intensity of the scattered light to the diameter and abundance of particles. This is performed using an aerosol generator of known size and optical properties. Thus, an OPC is defined by three parameters: the wavelength of the incident light, the scattering angle, and the number of particle-size intervals (24 in the case of the AlphaSense OPC-N3) [19]. The OPC-N3 counts the particles and creates a size distribution. The mass concentration is then obtained using an internal algorithm (black-box type) that uses the refractive index, particle density, and a weighting factor [24].

#### 2.5.2. Calibration by Means of Neural Network

In general, the aim of the calibration process is to find a function (*f*) that returns the concentration of each pollutant from the raw data of the electrodes (*S_WE_* and *S_AE_*; for simplicity, *WE* and *AE*) and environmental parameters such as temperature, relative humidity, pressure, and wind speed and direction.

In this work, the variables used as the inputs for this calibration function were the electrodes of each pollutant (*WE_i_* and *AE_i_*) (*i* = NO_2_, O_3_) and the temperature and relative humidity:

(2) $$f\left(WE_{i},\;AE_{i},\;T,\;RH\right)=\left[Pollutant\right]$$

An artificial neural network was used to calculate this calibration function. Neural networks are a fundamental tool in the field of low-cost-sensor calibration [25,26,27,28]. In a previous work [29], we tested this type of algorithm against other common techniques and concluded that neural networks performed the best.

A multilayer perceptron was designed with two hidden layers and two inputs per layer. The activation function of the neurons in the hidden layers was a rectified linear unit (ReLU). These parameters were chosen in such a way as to optimize the results. The process was performed using Python 3.8, making use of the scikit-learn 0.24.1 package.

Regarding the OPC-N3 particle sensor, the problems that arise when using it outdoors were exhaustively detailed in [7,24,30], highlighting the effect of temperature and the strong dependence on relative humidity. Relative humidity is especially relevant, since particles of suspended matter absorb part of the humidity in the air, which increases their size and modifies their refractive index, interfering with the sensor reading. In addition, it is important to highlight that because the OPC-N3 has a fan at the inlet, and because this fan is used to drive the flow to the gas sensors, a malfunction of the fan could affect both the particulate sensor and the two gas sensors. The status of the fan was monitored in all tests and will be discussed in the next section.

To solve the main problems of the OPC, another neural network was used. The inputs were the values returned by the OPC-NC particle sensor (*PM*_1_, *PM*_2_._5_, and *PM*_10_) and the temperature (*T*) and relative humidity (*RH*) as variables to describe the environment and the input flow rate (*FR*):

(3) $$f\left(PM_{i},\;T,\;RH,\;FR\right)=\left[PM\right]$$

For the gas and particle sensors, the calibration dataset was divided into training and test sets. The training set consisted of 60% of the data from the calibration campaign, and the test set contained the remaining 40%.

#### 2.5.3. Model Evaluation

In order to evaluate the effectiveness of the neural network in calculating the actual concentrations of pollutants, different metrics were calculated: slope, MAE, MSE, and coefficient of determination. Their expressions are as follows:

(4) $$\mathrm{Mean}\;\mathrm{absolute}\;\mathrm{error}\;(\mathrm{MAE})(y,\hat{y})=\frac{1}{n_{\mathrm{samples}}}\cdot \overset{n_{\mathrm{samples}}-1}{\underset{i=0}{\sum }}|y_{i}-\hat{y_{i}}|$$

(5) $$\mathrm{Root}\text{-}\mathrm{mean}\text{-}\mathrm{squared}\;\mathrm{error}\;(\mathrm{MSE})(y,\hat{y})=\sqrt{\frac{1}{n_{\mathrm{samples}}}\cdot \overset{n_{\mathrm{samples}}-1}{\underset{i=0}{\sum }}{(y_{i}-\hat{y_{i}})}^{2}}$$

(6) $${\mathrm{Coefficient}\;\mathrm{of}\;\mathrm{determination}\;(R}^{2})(y,\hat{y})=1-\;\frac{\sum_{i=0}^{n}{\left(y_{i}-\hat{y_{i}}\right)}^{2}}{\sum_{i=0}^{n}{\left(y_{i}-\bar{y_{i}}\right)}^{2}}$$

where $y_{i}$ is the real value of the pollutant for the *i*th sample (given by the reference equipment), $\hat{y_{i}}$ is the calculated value for that sample (given by the neural network), and $\bar{y_{i}}$ is the mean value of $y_{i}$.

### 2.6. Air-Quality Index

As mentioned above, the raw data collected from an LCS are generally inaccurate and not reproducible. For this reason, the use of an LCS is not a straightforward choice for making accurate measurements without refinement. However, it is an interesting tool for informing citizens about the overall levels of pollution that may be present in the environment. For this reason, in this work, the data collected during the cycling routes were processed and translated into different air-quality levels as stipulated by legislation.

Spanish legislation [31,32], based on recommendations of the European Environment Agency, includes a methodology that allows the calculation of the air-quality index from data of the official reference equipment. This index is divided into different levels with respective recommendations.

This system recommends reducing outdoor activity at level 4 for the general population and eliminating it completely at level 6. For people who belong to a risk group or are particularly sensitive to the effects of pollution, reducing outdoor activities is recommended at level 3 and eliminating them at level 5.

**Figure 3:**
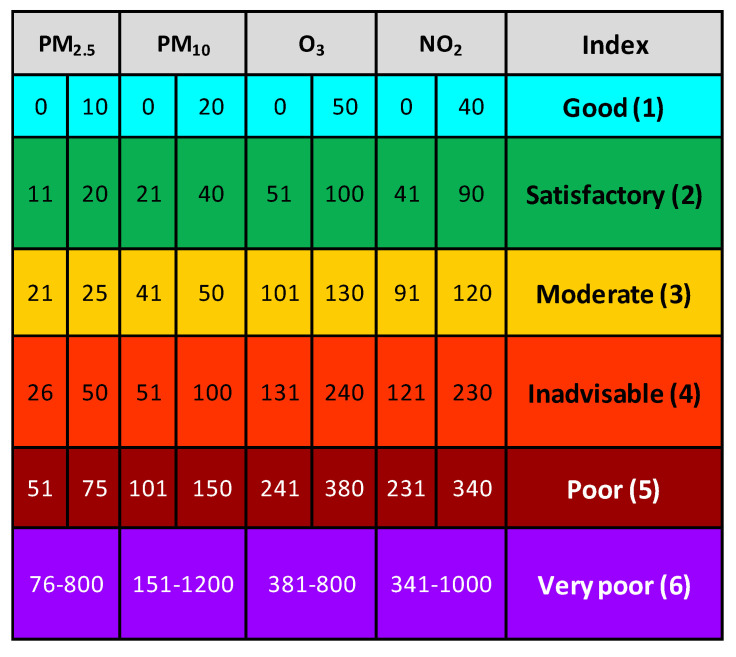
Air–quality levels (μg/m^3^) established by Spanish legislation.

According to the regulation, the air-quality index at a given time corresponds to the index with the highest value among the four pollutants (PM_2_._5_, PM_10_, O_3_, and NO_2_). To obtain this index, the regulation has established a method that involves 8-h-average data, which would require long measurement campaigns. This procedure was not practical for the purpose of this work. Therefore, to calculate the air-quality index at any time on a cycling route, the data were preprocessed, calibrated, and averaged to 1 point/5 s in order to obtain a more manageable resolution for users. These data were then compared to the values in Figure 3, thus obtaining an index for each pollutant.

In addition to the four pollutants mentioned above, SO_2_ is also included in this regulation; however, for this work it was decided not to include a sensor to measure this gas since it is found in very low concentrations in the study area [33].

In this work, the values in Figure 3 were used as a reference to determine the air-quality index at particular moments in order to inform users of the pollution status. However, it should be emphasized that the procedure by which the concentrations of pollutants were calculated is not exactly the same as the one indicated in the regulations [31,32]; therefore, this index will not correspond to the official one. The air-quality index calculated and shown in the following section should be understood as an index that allows users of the device to have immediate but approximate information on the state of the air quality at a local level and a specific time, given the data collected by the LCS.

## 3. Results and Discussion

### 3.1. Calibration and Validation Measurements

The calibration and validation processes applied to both devices are detailed below. For simplicity, only the information related to BEC01 is shown. Once the calibration and validation process has been explained, the results of the two devices will be shown for comparison.

**Figure 4:**
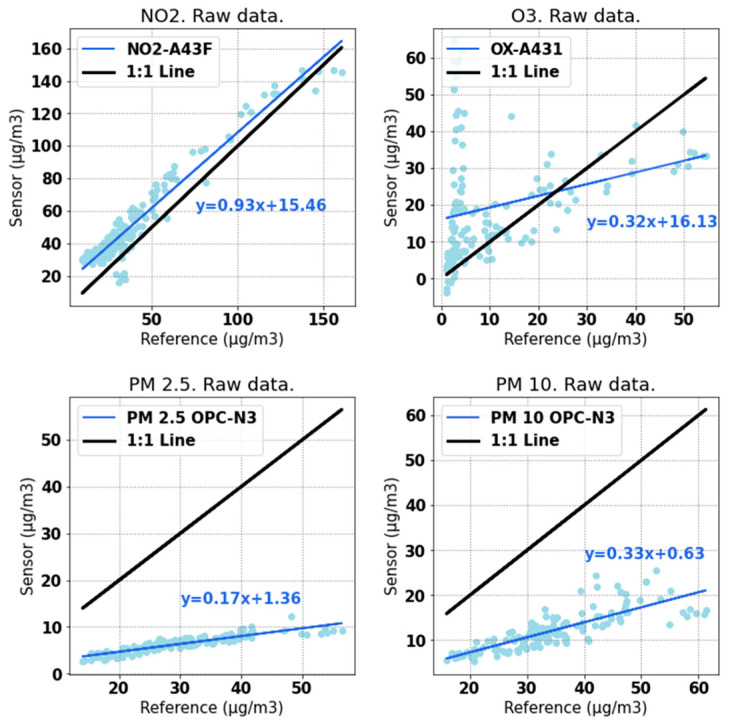
Scatter plots of values (μg/m^3^) from sensors vs. reference.

First, Figure 4 shows scatter plots comparing the raw data (i.e., not calibrated) collected by the device with data collected by the reference equipment, when both were collocated in parallel. It can be seen that the NO_2_-A43F sensor performed adequately even without being calibrated. The determination coefficient for this pollutant is 0.79 and the slope of the least squares line is 0.93, which are both very close to 1. This is not true for the ozone sensor, OX-A431, for which the coefficient of determination is R^2^ = −1.15 and the slope is 0.32. This difference between NO_2_ and O_3_ is mainly attributed to the fact that nitrogen dioxide was present in much higher concentrations than ozone during the period when the device was placed next to the reference station, since the calibration was carried out in winter when ozone is present in lower concentrations. In addition, as shown below, the ozone sensor showed a strong cross-sensitivity to NO_2_, which was not a problem with the reference station.

The OPC-N3 sensor shows a linear response with respect to the reference station, with both showing similar trends for PM_10_ and PM_2_._5_, but it returned lower values than the reference. The slope is 0.17 for PM_2_._5_ and 0.33 for PM_10_, which indicates low sensitivity. There is no significant offset error in OPC-N3, especially if we consider the non-calibrated LCS. Given that the fan incorporated in this sensor acts as an input to the other sensors, the performance of the OPC-N3 was studied in depth, as described later in this section.

**Figure 5:**
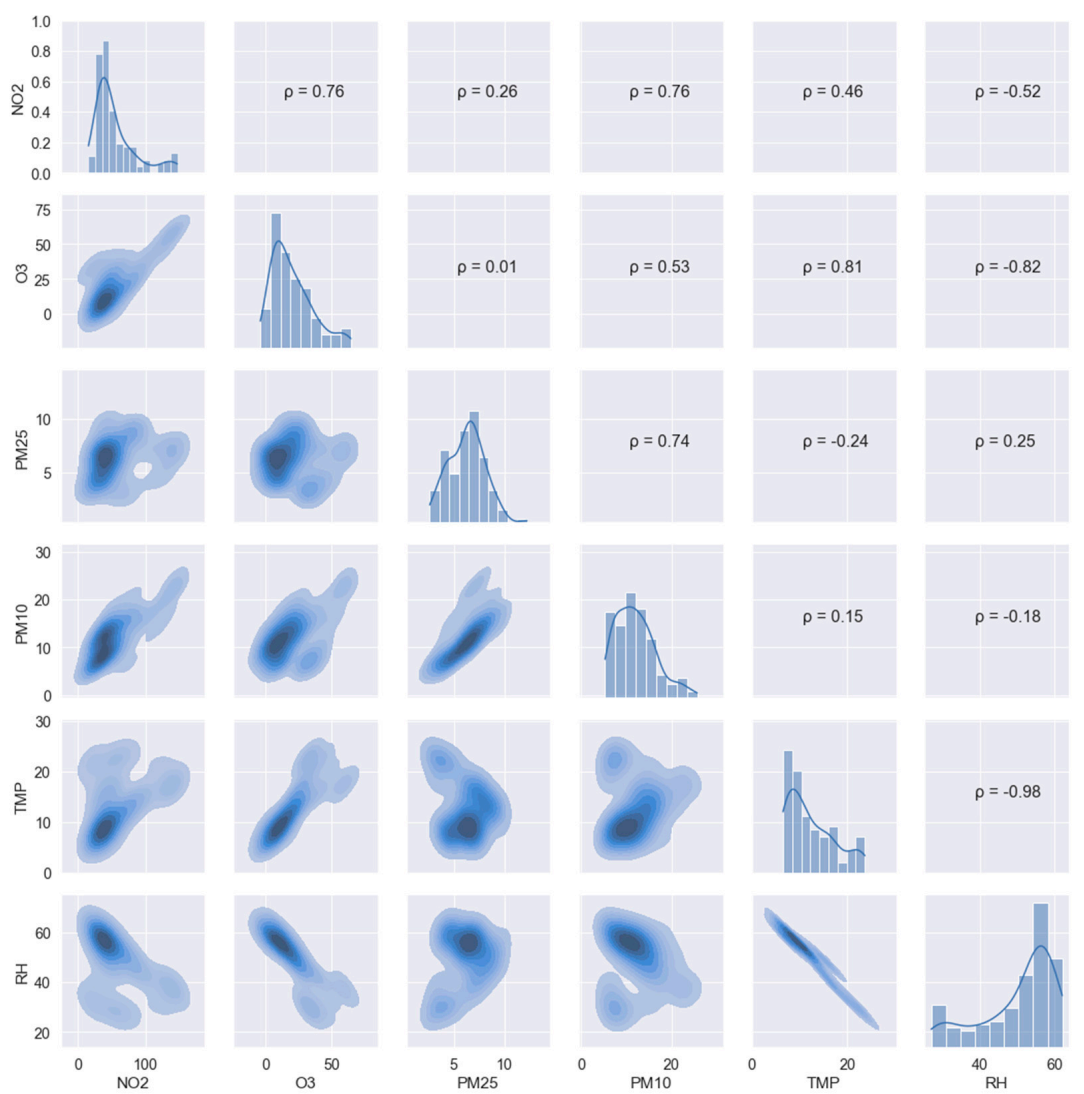
Data distribution and correlation among variables. Distribution of data is shown on the diagonal, with Pearson correlation coefficient above and kernel-density-estimator plot below. Measurement unit is micrograms per cubic meter (μg/m^3^).

Figure 5 shows the relationship between all pollutants monitored by the device along with the variables that characterize the environment (relative humidity and temperature). Thanks to this graph, very relevant information can be obtained on how these variables are related.

First, as mentioned above, the ozone sensor and nitrogen-dioxide sensor readings are closely correlated (ρ = 0.76) due to cross-sensitivity. This fact was exploited in the calibration of the sensors by adding the NO2-A43F electrode information to the O_3_ calibration and adding the OX-A431 electrode information to the NO_2_ calibration.

A strong dependence on temperature and relative humidity can also be observed, especially in the case of O_3_ (ρ = 0.81 and ρ = −0.82, respectively). No extreme values of temperature or relative humidity were reached during the time of measurement in parallel with the reference. The temperature ranged from 5.5 to 23.7 °C, while the humidity ranged from 29.9 to 62.0%, so these values always remained within the ranges established by the manufacturer.

#### OPC-N3 Results

**Figure 6:**
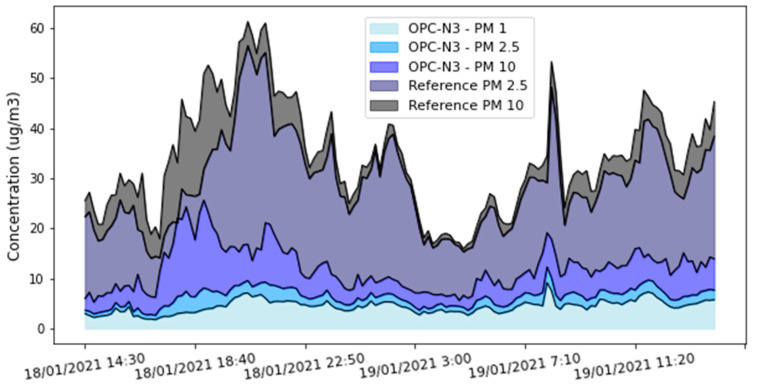
Time series of PM measured by reference and OPC-N3.

Next, the performance of the OPC-N3 particulate sensor was studied, as well as the inlet and outlet of the manifold that circulates air to the sensors. First, Figure 6 shows the concentrations of PM_2_._5_ and PM_10_ measured by the reference equipment along with the concentrations of PM_1_, PM_2_._5_, and PM_10_ measured by the particulate-matter sensor. It can be seen that the sensor follows the trend of the reference station but records lower values.

There are several possible explanations for this phenomenon. First, hygroscopic growth, i.e., the increased optical size of particles due to the absorption of ambient humidity, would imply that the OPC is overestimating the real particle size, classifying them as larger than 10 microns, thus underestimating the concentrations of PM_2_._5_ and PM_10_ [34].

**Figure 7:**
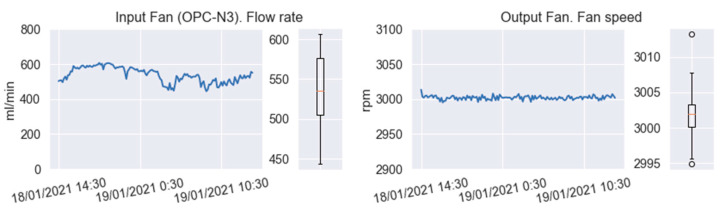
OPC-N3 inlet and outlet fans of the air manifold.

Another possible reason is the placement of the device during the measurement period in parallel with the reference equipment. The air inlet to the reference station was placed vertically, while that of the device was in a horizontal position, which means that the wind direction could have affected the air flow entering the sensors. Figure 7 shows the status of the inlet fan (in the OPC-N3) and the outlet fan. As noted by the manufacturer of the particle sensor [35], the speed of the inlet fan can vary as a consequence of wind action. Figure 7 also shows the status of the outlet fan. The manufacturer states that for proper operation, the output-fan speed must be 3000 ± 100 rpm, so the device was within the established limits. It is important to highlight that the problem of the position of the OPC-N3 sensor with respect to the wind direction did not apply during the bicycle routes, since in such cases the sensor inlet was located on one side, which means the forward movement of the bicycle did not generate a gas-flow problem.

Based on the comparison between the NO_2_ and O_3_ measured by the device and by the reference (Figure 4), it does not seem that the LCS underestimated the concentrations of these two gases. Therefore, since the input flow rate could have affected the OPC measurements, but not the gas-sensor measurements, the flow rate was added as an input to the neural network that was used to calibrate the particulate-matter sensor.

### 3.2. Model Performance

**Figure 8:**
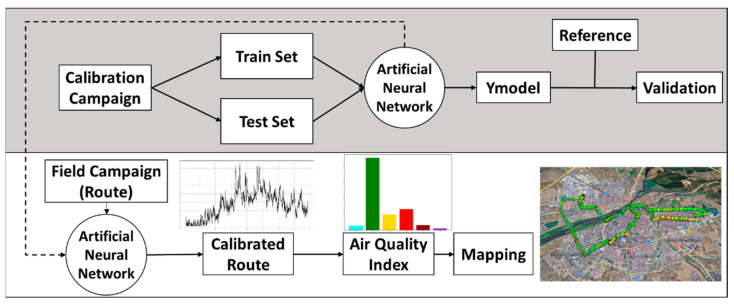
Calibration and measurement process flowchart.

The measurements collected by the device along with those of the reference equipment when both were measuring in parallel were used to calibrate the sensors. This calibration was subsequently used to correct the data from the bicycle routes, as shown in Figure 8.

As mentioned in Section 2.5.2, this calibration was performed by means of a neural network. The results obtained by using this algorithm are shown in Table 1. It can be seen that the calibration process improved all metrics that were chosen to evaluate the model. Specifically, the slope of the regression line improved significantly for O_3_, PM_2_._5_, and PM_10_ and remained at a value close to unity for NO_2_. Both the mean absolute error and root-mean-squared error were significantly reduced in all four cases. Finally, the coefficient of determination ranged from −5.72 to 0.79 to more than 0.83 for all four pollutants.

**Figure 9:**
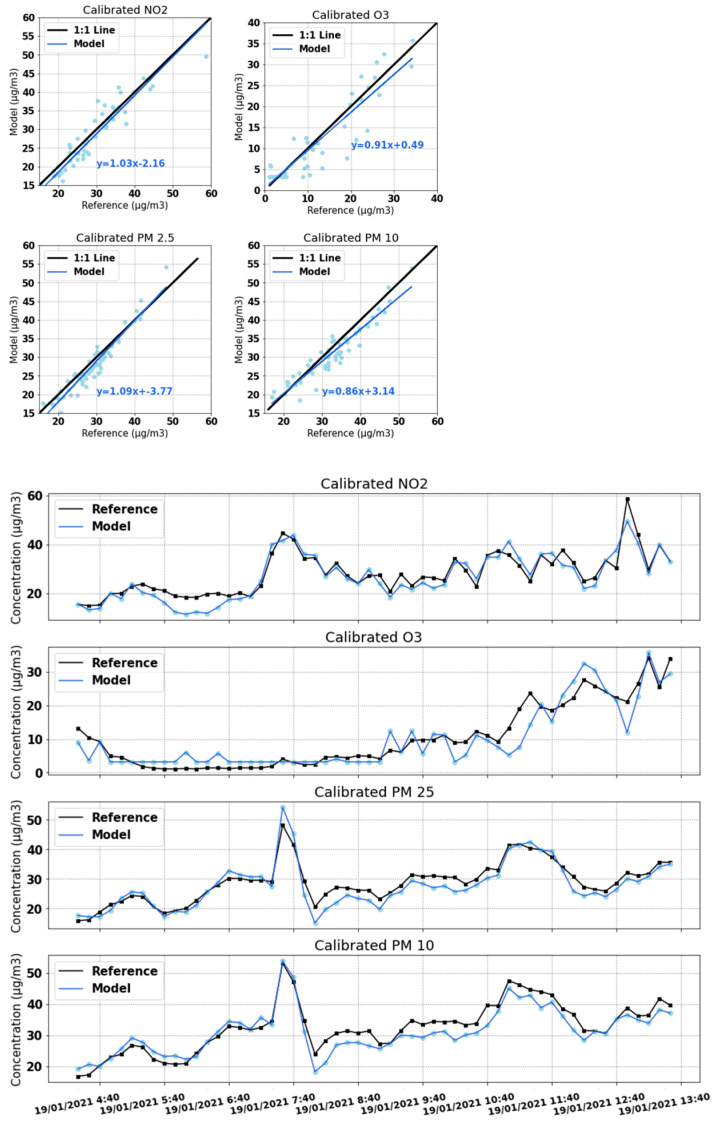
Calibrated data for NO_2_, O_3_, PM_2_._5_, and PM_10_. Measurement unit is micrograms per cubic meter (μg/m^3^).

Figure 9 shows, at the top, scatter plots that compare the values of the sensors after being calibrated with the values measured by the reference. Below are the time series of the four pollutants for the time period used as a test.

### 3.3. Comparison between Devices

**Figure 10:**
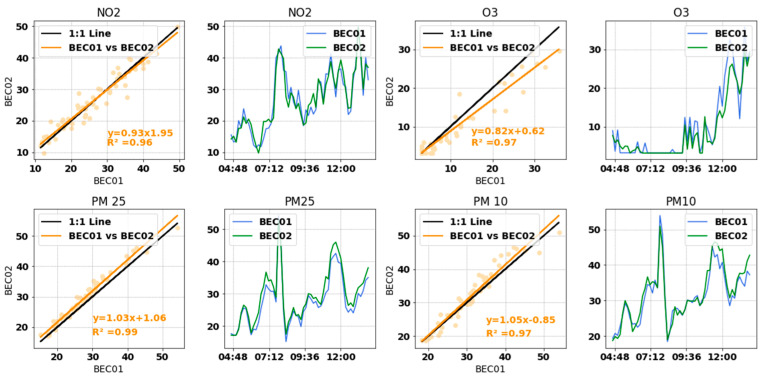
Scatter plots and temporal evolution of BEC01 and BEC02 after calibration. Orange indicates points and least-squares line of BEC01 vs. BEC02; blue represents BEC01 and green represents BEC02. Measurement unit is micrograms per cubic meter (μg/m^3^).

Figure 10 and Table 2 show that once the calibration was applied to the devices (BEC01 and BEC02), both devices collected the same readings. In all four cases, the coefficient of determination was above 0.96. Ozone showed the lowest repeatability among pollutants. This is mainly attributed to the quite low concentration of O_3_ (minimum concentration 3.13 μg/m^3^), as expected in winter, so the LCS can barely measure it. On the other hand, the OPC-N3 in BEC01 performed almost exactly the same as in BEC02.

### 3.4. Bicycle Routes

The next stage of this work consisted of processing the multiparametric data from the three bicycle routes to obtain simplified information on the state of the pollutants. Other studies such as [12,36,37] calculated an air-quality index to express the concentration values in a way that could be understood by all users. In our case, once the calibration algorithm was trained and validated, it was applied to the data collected by the device on the three routes in the city of Badajoz as described above. An air-quality index based on multiparametric data provides richer and more usable information about air pollution based solely on a single parameter with respect to other reported strategies, e.g., the work of Gao et al. that used the dynamic measurement of PM_2_._5_ by sensors attached to buses [38] or of Cheng et al. on the performance of a network of static PM_2_._5_ sensors [39]. Other works, such as [40], monitored air quality using mobile equipment coupled with bicycles, but they focused mainly on ultrafine particles and black carbon, which are not the main factors responsible for worsening air quality according to Spanish legislation. Finally, we obtained better numerical results compared to the previously mentioned works (RMSE = 2.59 vs. >96.69 in [39], and R^2^ > 0.83 vs. in the range (0.64–0.95) in [40]).

**Figure 11:**
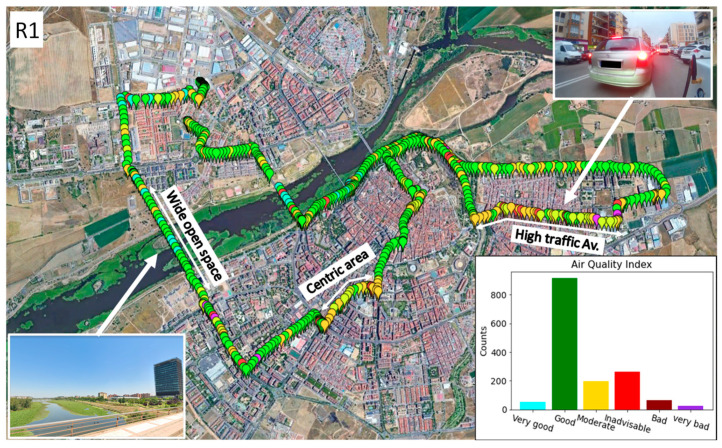
Route 1, Badajoz (Spain), on 22 January 2021. Most relevant zones in terms of air-quality index are shown, and bar chart representing frequency of appearance of each air-quality level.

Once the air-quality index was obtained, using the GPS data collected by the device, the results of the bicycle routes could be mapped. Figure 11 shows the air-quality index along the route taken on Friday 22 January 2021 (R1), between 17:10 and 18:30. In this case, it is shown with 1 point/5 s resolution. It is observed that during most of the route, the index was between level 1 (very good) and level 3 (moderate). The areas of the city where the index was most favorable coincide with areas near the river, which had large open spaces and low traffic at that time of day. On the other hand, on crowded avenues and in downtown areas, the air-quality index exceeded level 4 (unadvisable). These points coincide with specific times when the bicycle was moving behind another vehicle because of traffic.

**Figure 12:**
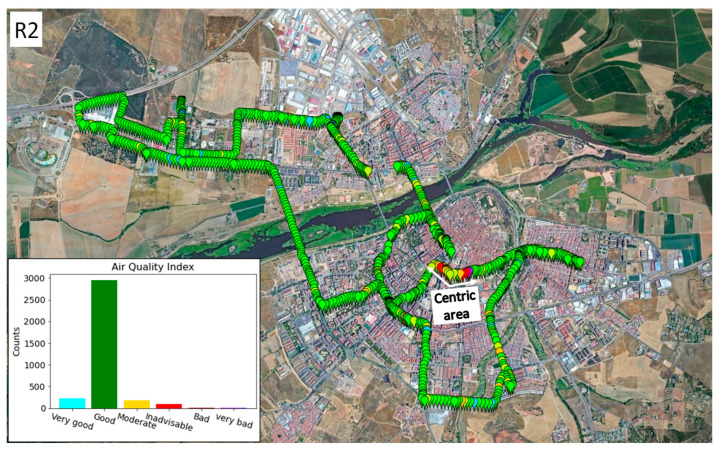
Route 2, Badajoz (Spain), on 24 January 2021.

The second route (Figure 12) was carried out on Sunday, 24 January 2021, between 16:00 and 18:00. It can be seen that the impact of traffic was much lower than on the first route. Only one area where the air quality was unadvisable or worse can be seen, which was on a central avenue of the city where there was quite a lot of traffic even though it was Sunday afternoon.

**Figure 13:**
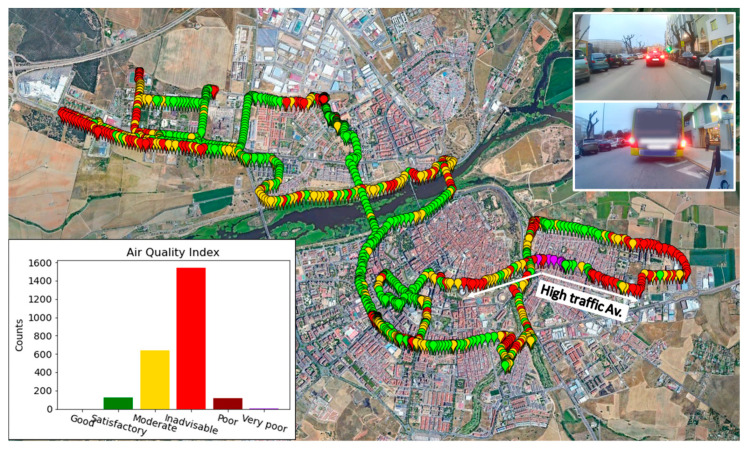
Route 3, Badajoz (Spain), on 18 January 2021.

Finally, the third route (Figure 13) took place on Thursday, 28 January 2021, between 7:50 and 9:50. At this hour on a workday, it is typical to encounter a lot of traffic in the city. This is reflected in the air-quality index, as the sensors were greatly affected by the gases emitted from nearby vehicles. At this time, the air-quality index was mostly between levels 3 and 4 (moderate and inadvisable). At no time did it drop below level 2, unlike the two previous routes, which were carried out in the afternoon amid much lower traffic density.

## 4. Conclusions

An electronic prototype was developed to monitor air quality in motion and in real time. It is based on low-cost electrochemical sensors and was designed to be coupled to a bicycle. This system obtains reliable information on concentrations of NO_2_, O_3_, PM_2_._5_, and PM_10_ in order to inform bike users and citizens.

The device has a novel design: it takes advantage of the OPC-N3 air-supply pump as an input for the two gas sensors, NO2-A43F and OX-A431. However, this design requires paying special attention to the OPC-N3, since a malfunction in the air-supply pump would affect not only the PM measurements but also the NO_2_ and O_3_ measurements. The inlet variables were examined to ensure their correct operation, and the airflow to the sensors was incorporated as an additional input to the calibration algorithm.

Parallel measurements with the reference show some initial deviations of the LCS. These deviations were corrected by the calibration algorithm. Calibration by a neural network allows the sensor accuracy to be increased, with a coefficient of determination up to 0.85.

On the other hand, comparing the two devices made it possible to ensure repeatability. We conclude that there are no meaningful differences between BEC01 and BEC02.

The air-quality-index mapping described in this work allows us to inform citizens about air quality in a simple, easy-to-understand way. Preliminary field testing of the device during short-term cycling routes in urban areas during wintertime showed that the device can provide information about which areas of the city have better air quality, which days of the week have differences in the air-quality index, and at what times the impact of traffic is more severe, making the device a useful tool for citizens in addition to traditional instruments. Work is in progress to improve the quality testing of the system by conducting additional campaigns of longer duration and including different meteorological scenarios, with the goal of constructing a model of spatial distribution and temporal evolution of air pollutants along urban cycling routes.

## Figures and Tables

**Table 1:** Metrics of artificial neural network performance as a calibration algorithm. Measurement unit is micrograms per cubic meter (μg/m^3^).

Pollutant	Metric	Before Calibration	After Calibration
NO_2_	Slope	0.93	1.03
	MAE	13.94	2.80
	RMSE	15.11	3.54
	R^2^	0.79	0.83
O_3_	Slope	0.32	0.91
	MAE	12.62	2.75
	RMSE	19.50	3.74
	R^2^	−1.15	0.84
PM_2_._5_	Slope	0.17	1.09
	MAE	23.05	6.73
	RMSE	22.70	2.59
	R^2^	−5.72	0.85
PM_10_	Slope	0.33	0.86
	MAE	22.18	2.56
	RMSE	23.45	3.07
	R^2^	−3.72	0.85

**Table 2:** Repeatability between BEC01 and BEC02 before and after calibration. Measurement unit is micrograms per cubic meter (μg/m^3^).

Pollutant	Metric	After Calibration
NO_2_	Slope	0.93
	Intercept	1.95
	R^2^	0.96
O_3_	Slope	0.82
	Intercept	0.62
	R^2^	0.97
PM_2_._5_	Slope	1.07
	Intercept	1.06
	R^2^	0.99
PM_10_	Slope	1.05
	Intercept	0.85
	R^2^	0.97
